# Association between fractional exhaled nitric oxide, sputum induction and peripheral blood eosinophil in uncontrolled asthma

**DOI:** 10.1186/s13223-018-0248-7

**Published:** 2018-05-23

**Authors:** Jie Gao, Feng Wu

**Affiliations:** Department of Respiratory Medicine, The Third People’s Hospital, Guangzhou Medical College, 1# Xuebei Ave., Huizhou, 516002 Guangdong China

**Keywords:** Asthma, Sputum, FeNO, Blood, Eosinophil, Neutrophil, BHR, Bronchial reversibility

## Abstract

**Background:**

The fractional exhaled nitric oxide (FeNO) and blood eosinophils are biomarkers of eosinophilic airway inflammation used in the diagnosis and management of asthma, although induced sputum is the gold standard test for phenotypic asthma. Nevertheless, the clinical application of the correlation between sputum eosinophils, FeNO and blood eosinophils is controversial.

**Objective:**

To investigate the clinical application of the correlation between sputum eosinophils, FeNO and blood eosinophils with uncontrolled asthmatic patients. It also examined the relationships between these biomarkers in bronchial reversibility and bronchial hyper-responsiveness (BHR).

**Methods:**

This study evaluated 75 uncontrolled asthmatic patients (symptom control and future risk of adverse outcomes). All patients underwent the following on the same day: FeNO, spirometry, BHR or bronchodilator reversibility, sputum induction and blood collection. Eosinophilic airway inflammation was defined as sputum eosinophils ≥ 2.5% or FeNO levels ≥ 32 parts per billion (ppb).

**Results:**

A significant positive relationship was between percentage of sputum eosinophils and FeNO (r = 0.4556; p < 0.0001) and percentage of blood eosinophils (r = 0.3647; p = 0.0013), and a significant negative correlation was between percentage of sputum neutrophils and FeNO (r = − 0.3653; p = 0.0013). No relationship between FeNO and percentage of blood eosinophils (p = 0.5801). ROC curve analysis identified FeNO was predictive of sputum eosinophilia [area under the curve (AUC) 0.707, p = 0.004] at a cutoff point of 35.5 ppb (sensitivity = 67.3%, specificity = 73.9%). Percentage of blood eosinophils was also highly predictive with an AUC of 0.73 (p = 0.002) at a cut-off point of 1.5%, sensitivity and specificity were 61.5 and 78.3%, respectively. Although the sputum neutrophil percentage was correlated with FeNO, ROC curve of these parameters did not show useful values (AUC = 0.297, p = 0.003; AUC = 0.295, p = 0.021).

**Conclusions and clinical relevance:**

Blood eosinophils and FeNO can accurately predict eosinophilic airway inflammation in uncontrolled asthmatic patients. FeNO is poor surrogates for sputum neutrophils and blood eosinophils. The FeNO level and blood eosinophils, which determine an optimal cutoff for sputum eosinophilia, need more studies.

## Background

Asthma is a heterogeneous disease, characterized by the history of variable respiratory symptoms such as wheeze, shortness of breath, chest tightness and cough, usually together with variable expiratory airflow limitation. This was reached by consensus, recommended by the 2017 Global Initiative for Asthma (GINA) [1]. It has been recognized that among asthmatic patients there are clusters of demographic, clinical and/or pathophysiological characteristics, which has been called “asthma phenotypes” [1, 2]. However, to date, no strong relationship has been found between specific pathological features and particular clinical or treatment responses [3]. When examining the airway inflammation using sputum analysis, patients with asthma can be classified in four different inflammatory phenotypes. Based on the percentage of eosinophils and neutrophils in sputum, it was to define four airway inflammatory subgroups: eosinophilic asthma, neutrophilic asthma, mixed granulocytic asthma and paucigranulocytic asthma [4]. There is recent evidence from prospective clinical studies that airway inflammatory phenotype can help to optimise therapy and disease outcome. Eosinophilic asthma responds well to anti-inflammatory treatment with steroids, non-eosinophilic asthma shows little or no response [5]. This suggests that biomarkers of inflammation could be considered in identifying and monitoring of asthmatic patients in clinical practice, such as the titration of steroid treatment.

Airway inflammatory phenotype can be measured through the airway noninvasively by induced sputum analysis [2, 4] and fractional exhaled nitric oxide (FeNO) [6]. Both are considered as a direct, reliable, sensitive, simple, and repeatable method of assessing inflammatory phenotypes, widely used in clinical practice. Peripheral blood eosinophils has also been studied as another potentially biomarker; it is associated with the patient’s future risk for exacerbations [7, 8], the risk factor for developing fixed airflow limitation [9], the diagnosis of eosinophilic asthma [10]. In 2016, the normal reference values of induced sputum cytology in China, defined as sputum eosinophils ≥ 2.5%, and increases in FeNO level ≥ 32 ppb, were identified as airway eosinophilia [11]. Nevertheless, the clinical application of the correlation between FeNO levels, sputum eosinophils and peripheral blood eosinophils is controversial.

We conducted a retrospective study to (1) evaluate the correlation between sputum eosinophils, FeNO level and peripheral blood eosinophils in patients with asthma (2) to determine the accuracy of these biomarkers as indicators of airway inflammatory phenotypes in these patients (3) assess the relationship between these biomarkers, bronchial hyper-responsiveness (BHR) and bronchodilator reversibility in asthma.

## Methods

### Study design and participants

We conducted a retrospective study on a series of 65 patients with asthma visiting in The Third People’s Hospital of Guangzhou Medical College in Huizhou from April 2016 to June 2017. Asthma patients were diagnosed according to a clinical history of wheezing, cough, chest tightness or shortness of breath, as well as the presence of bronchial hyper-responsiveness or bronchodilator reversibility, based on the 2016 of the Chinese national Guidelines on Diagnosis and Management of Asthma [12]. Included asthma patients received initial diagnosis and were uncontrolled stage. The level of asthma control was defined by asthma symptoms control (in the past 4 weeks, has the patient had daytime asthma symptoms more than twice/week and/or any night waking due to asthma and/or any activity limitation due to asthma and/or reliever needed for symptoms’ more than twice/week) and future risk of adverse outcomes (in the past 4 weeks, has the patient had FEV_1_% less than normal predicated value and/or any severe exacerbation due to asthma). Uncontrolled asthmatic patient had equal to or greater than 3 features as the above.

Inclusion criteria were any patients with asthma aged ≥ 18 years who agreed to undergo detailed investigation. All the patients who had successful FeNO, lung function and sputum induction were included in the study. Data were collected during regular clinical practice and medical procedures. Their demographic and functional characteristics are summarized in Tables 1, 2 and 3.

**Table 1:** Patient demographics and baseline characteristics

Demographic parameter	All participants (N = 75)
Age, years, median	61 (53, 71)
Range	18–85
Males, n (%)	42 (56)
Race, n (%)	
Chinese	75 (100%)
Mean height, cm (SD)	160 (8.78)
Range	136–174
Mean weight, kg (SD)	59 (9.29)
Range	44.5–84
Mean body mass index, kg/m^2^ (SD)	23.2 (3.61)
Range	16.71–34.22
Smokers, n (%)	32 (42.7)

N, total population; n, sub-group population; SD, standard deviation

**Table 2:** Spirometry results for the patients

Variable	All participants (N = 75)
FVC (L), mean (SD)	2.94 (0.86)
FVC% predicted, mean (SD)	97.03 (13.96)
FEV1 (L), mean (SD)	1.99 (0.57)
FEV1% predicted, mean (SD)	81.77 (11.86)
FEV1/FVC (%), mean (SD)	68.33 (8.54)
PEF (L/min), mean (SD)	5.33 (1.76)
PEF% predicted, mean (SD)	78.45 (17.64)
MMEF, mean (SD)	1.36 (0.5)
MMEF% predicted, mean (SD)	40.46 (18.25)
MEF50% (L/s), mean (SD)	1.76 (0.76)
MEF50% predicted, mean (SD)	47.55 (20.86)
MEF25% (L/s), mean (SD)	0.59 (0.26)
MEF25% predicted, mean (SD)	45.34 (19.62)

FVC, forced vital capacity; FEV1, forced expiratory volume in 1 s; PEF, peak expiratory flow; MMEF, maximum mid-expiratory flow; MEF, maximal expiratory flow

**Table 3:** FeNO level, induced sputum and peripheral blood result for the patients

Variable	All participants (N = 75)
FeNO level, ppb	38 (20, 72)
Eosinophil (%) in sputum	4.2 (1.1, 15.7)
Neutrophil (%) in sputum, mean (SD)	78.51 (17.3)
White blood cell (× 10^9^/L)	7.6 (6.4, 9.9)
Eosinophil (%) in peripheral blood	0.1 (0, 0.3)
Eosinophil count in peripheral blood (× 10^9^/L)	1.4 (0.4, 3.2)

Values are expressed as median (inter quartile range) or mean (SD)

Exclusion criteria were any patients had a history with chronic obstructive pulmonary disease (COPD), or previous doctor-diagnosed asthma-COPD overlap [(ACO), COPD/ACO was distinguished according to the 2017 recommendation of GINA, on the basis of chronic respiratory symptoms and post-bronchodilator FEV_1_/FVC < 0.7 or FEV_1_/FVC < 0.7 after treatment]. Patients have used any oral or/and inhaled corticosteroid in the previous 12 weeks. Patients had a confounding pulmonary comorbidity such as a pulmonary tuberculosis, an interstitial lung disease, a lung cancer or a pulmonary infection. Patients had a cognitive impairment that may affect the collaboration or comprehension of the study.

### Ethics statement

The Institutional Review Board of the Third People’s Hospital of Guangzhou Medical College in Huizhou approved the study protocol and absolved the need for written informed consent from patients as the study was a retrospective study, personal identification data were anonymized.

### Assessments and study procedures

On the same day the following tests or determinations were performed: FeNO test, pulmonary function test (PFT), BHR test or bronchodilator reversibility test, induced sputum and routine blood test. Clinical variables were recorded for the participants.

FeNO: FeNO level was measured before PFT according to the guidelines in the user manual training on the NO electrochemical equipment (NIOX VERO; Aerocrine AB, Solna, Sweden). Patients were required to refrain from eating, drinking, and smoking for at least 1 h prior to the FeNO measurement. Patients were instructed to inhale NO-free air to total lung capacity and immediately exhale fully into the device at a sustained flow rate of 50 mL/s for 6 or 10 s, which resulted in display of FeNO value 6. A significant increase in FeNO was considered if the FeNO value was equal to or higher than 32 parts per billion (ppb) [6].

PFT: Airway limitation was identified using lung function machine (MS-pneumo + aps; Jaeger, Friedberg, German) by an experienced technician according to the 2014 recommendations of the Chinese National Guidelines of Pulmonary Function Test. Percentage predicted values (%pred) were calculated based on reference values for healthy Chinese adults. All patients were required to undergo PFT in a reproducible way, and the best values were retained [13].

BHR test: PFT values were assessed prior to the methacholine challenge. Patients with a FEV1%pred < 60% were excluded from the BHR test (at baseline). The breath dosimeter method was used according to published guidelines from Chinese National Guidelines of Pulmonary Function Test. The test sequence included five steps: 0.9% NaCl only, 0.078, 0.312, 1.125 and 2.504 mg. Measure the FEV_1_ at about 60 s from the start of one to the start on the next inhalation from the nebulizer. Obtain an acceptable-quality FEV1 at each time point. Airway responsiveness was required to induce a 20% decrease in FEV1 (PD_20_), and the positive response was defined as PD_20_ ≤ 2.504 mg (between NS and 2.504 mg) [13].

Bronchodilator reversibility test: Patients were asked to inhale 400 μg salbutamol via a metered-dose inhaler after baseline evaluation, and PFT was repeated not less than 20 min. Three forced expiratory maneuvers were recorded. The positive response, which was defined as FEV_1_ > 12% and 200 mL after salbutamol inhalation, were obtained [13].

Sputum induction: Sputum was induced with hypertonic saline inhalation through ultrasonic atomizer. A single hypertonic saline (3%NaCl) was used. Patients were asked to inhale 400 μg salbutamol via a metered-dose inhaler 20 min before induction. Collected lower respiratory sputum portions of induced sputum were dispersed using 0.1% dithiothreitol in a water bath (37 °C) and oscillator 15 min before the 300 mesh nylon mesh filter. Subsequently, total cell count was centrifuged, smeared and stained (hematoxylin–eosin). A differential cell count was obtained from 400 cells under 400× microscope to identify the type of airway inflammation in patients with asthma. We defined percentage of sputum eosinophils ≥ 2.5% as abnormal [11].

Blood collection and analysis: Peripheral venous blood was measured using Automated Hematology Blood Analyzer (ABX Pentra DF120–1; ABX, France). The results of peripheral blood eosinophil percentage and absolute eosinophil count were obtained.

### Statistical analysis

Analysis of the data was performed using SPSS 19 (IBM Corporation, Armonk, NY, USA). Continuous variables are expressed as mean ± SD or median (interquartile range) for non-normal variables when appropriate, and categorical variables with the Chi square test. Non-normally distributed variables used the Mann–Whitney test. The relationship between FeNO, sputum eosinophils, blood eosinophils, BHR and bronchodilator reversibility was assessed using the Spearman’s rank correlation coefficient. Correlation between tests was performed by constructing receiver operating characteristic (ROC) curve. The optimal cutoff value was determined from the highest sum of sensitivity and specificity. Statistical significance was defined as p < 0.05.

## Results

### Characteristics of the patients

Patient demographic information is presented in Table 1. A total of 75 patients with uncontrolled asthma participated [42 (56%) males] in the study. The median age was 61 years with a range between 18 and 85 years. Smokers accounted for 42.7% of patients. All participants were Chinese. PFT results are reported in Table 2. Patients with uncontrolled asthma had a mean FEV1/FVC% of 68.33% and a mean FEV1%pred of 81.77%. The result of FeNO level, induced sputum and peripheral blood are reported in Table 3.

### Sputum induction

Eosinophilic airway inflammation (sputum eosinophilia ≥ 2.5%) was present in 52 participants. The characteristics of patients are shown in Table 4. Patients in sputum eosinophilia group compared with patients in sputum noneosinophilia group showed a significantly higher FeNO level (p = 0.011), in peripheral blood eosinophil percentage (p = 0.003) and absolute eosinophil count (p = 0.016).

**Table 4:** FeNO levels and peripheral blood result for the patients

Variables	Sputum eosinophilia (n = 52)	Sputum noneosinophilia (n = 23)	p-value
Age, years	58 (50, 66)	64 (59, 76)	0.224
Males, n (%)	30 (57.7)	12 (52.2)	0.657
Mean body mass index, kg/m^2^	23.3 (3.86)	22.98 (3.03)	0.476
Smokers, n (%)	23 (44.2)	9 (39.1)	0.68
Postbronchodilator FEV1% predicted	80.48 (8.57)	84.7 (17.02)	0.07
Postbronchodilator FEV_1_ (L)	2.06 (0.58)	1.81 (0.51)	0.548
Postbronchodilator FEV_1_/FVC (%)	68.12 (8.95)	68.82 (7.7)	0.297
FeNO level (ppb)	48 (25, 92)	24 (19, 47)	*0.011*
Neutrophil (%) in sputum	74.36 (18.11)	87.7 (10.55)	0.062
White blood cell (×10^9^/L)	7.3 (6.2, 9.6)	8.5 (6.5, 10.3)	0.192
Eosinophil (%) in peripheral blood	1.8 (0.6, 4.8)	0.5 (0.2, 1.8)	*0.003*
Eosinophil count in peripheral blood (×10^9^/L)	0.1 (0.1, 0.3)	0 (0, 0.2)	*0.016*

Sputum eosinophilia refers to sputum eosinophils ≥ 2.5%; sputum noneosinophilia refers to sputum eosinophils < 2.5%. Significant p value < 0.05

### FeNO

FeNO level equal to or higher than 32 ppb was present in 45 participants. The characteristics of patients are shown in Table 5. Patients in the two groups showed a meaningless result in eosinophilic cell in peripheral blood percentage and absolute count, but a significantly increased in the percentage of sputum eosinophils (p < 0.001).

**Table 5:** Sputum induction and peripheral blood result for the patients

Variables	FeNO ≥ 32 ppb (n = 45)	FeNO < 32 ppb (n = 30)	p-value
Age, years	58 (49, 65.5)	63.5 (58.5, 75.3)	0.386
Males, n (%)	26 (57.8)	16 (53.3)	0.704
Mean body mass index, kg/m^2^	22.62 (3.35)	24.07 (3.86)	0.396
Smokers, n (%)	21 (46.7)	11 (36.7)	0.391
Postbronchodilator FEV1% predicted	82.38 (12.68)	80.86 (10.64)	0.71
Postbronchodilator FEV1 (L)	2.12 (0.56)	1.78 (0.52)	0.875
Postbronchodilator FEV1/FVC (%)	68.22 (8.7)	68.5 (8.44)	0.953
Eosinophil (%) in sputum	9.89 (3.65, 20.53)	2.44 (0.59, 4.05)	< *0.001*
Neutrophil (%) in sputum, mean (SD)	74.15 (18.56)	84.9 (12.83)	0.076
White blood cell (×109/L)	7.6 (6.3, 10.3)	7.5 (6.4, 9.6)	0.841
Eosinophil (%) in peripheral blood	1.8 (0.3, 4.8)	1.3 (0.5, 2.93)	0.97
Eosinophil count in peripheral blood (×109/L)	0.1 (0, 0.3)	0.1 (0.08, 0.23)	0.889

Significant p value < 0.05

### Peripheral blood eosinophils

Eosinophilic percentage in peripheral blood ≥ 2% was present in 28 participants. The characteristics of patients are shown in Table 6. The percentage of sputum eosinophils (p = 0.001) and blood absolute eosinophil count (p < 0.001) were significantly higher in blood eosinophilia group. Nonetheless, FeNO level was not different between groups.

**Table 6:** FeNO level and sputum induction result for the patients

Variables	Blood eosinophilia (n = 28)	Blood noneosinophilia (n = 47)	p-value
Mean age, years	58 (52.3, 71.3)	62 (53, 71)	0.496
Males, n (%)	16 (57.1)	26 (55.3)	0.878
Mean body mass index, kg/m^2^	22.64 (3.15)	23.54 (3.85)	0.509
Smokers, n (%)	11 (39.3)	21 (44.7)	0.648
Postbronchodilator FEV1% predicted	83.29 (7.33)	80.87 (13.87)	0.23
Postbronchodilator FEV1 (L)	2.13 (0.59)	1.9 (0.54)	0.696
Postbronchodilator FEV1/FVC (%)	68.32 (8.02)	68.34 (8.92)	0.505
FeNO level, (ppb)	48 (25, 95)	35 (19, 65)	0.103
Eosinophil (%) in sputum	9.85 (3.52, 24.83)	3.6 (0.5, 10.3)	*0.001*
Neutrophil (%) in sputum, mean (SD)	71.37 (19.34)	82.66 (14.51)	0.151
White blood cell (×10^9^/L)	6.9 (6, 9.2)	7.9 (6.8, 10.3)	0.069
Eosinophil count in peripheral blood (×10^9^/L)	0.3 (0.2, 0.7)	0.1 (0, 0.1)	< *0.001*

Blood eosinophilia refers to peripheral blood eosinophils ≥ 2%; blood noneosinophilia refers to peripheral blood eosinophils < 2%. Significant p value < 0.05

### Associations between FeNO level, sputum induction, peripheral blood eosinophils, BHR and bronchodilator reversibility

**Fig. 1:**
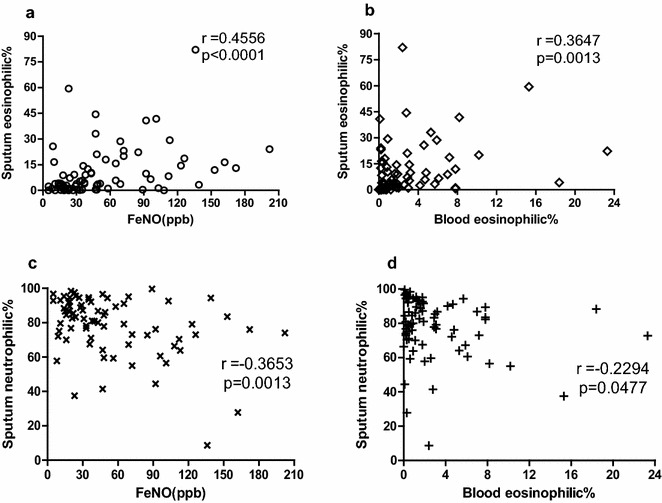
Scatter plots of correlation between sputum eosinophil/neutrophil percentage, FeNO level and peripheral blood eosinophil percentage. **a** Correlation between sputum eosinophil percentage and FeNO level. **b** Correlation between sputum eosinophil percentage and blood eosinophil percentage. **c** Correlation between sputum neutrophil percentage and FeNO level. **d** Correlation between sputum neutrophil percentage and blood eosinophil percentage

A significant positive relationship was observed between percentage of sputum eosinophils and FeNO level (r = 0.4556; p < 0.0001) (Fig. 1a) and percentage of blood eosinophils (r = 0.3647; p = 0.0013) (Fig. 1b), and a significant negative correlation between percentage of sputum neutrophils and FeNO level (r = − 0.3653; p = 0.0013) (Fig. 1c). We also found weaker but significant correlations between percentage of sputum neutrophils and percentage of blood eosinophils (r = − 0.2294; p = 0.0477) (Fig. 1d). No relationship between FeNO level and percentage of blood eosinophils (p = 0.5801).

Evidence of variable airflow obstruction was observed in 75 participants, 44 of whom had airway hyper-responsiveness based on the methacholine challenge results, and 31 had bronchodilator reversibility. There were no significant relationship between PD20 and percentage of sputum eosinophils (p = 0.2505), PD20 and FeNO level (p = 0.0955), PD20 and blood eosinophil percentage/count (p = 0.4517; p = 0.4933; respectively). There were also no significant relationship between ∆FEV1 (∆, improvement in FEV1 after 400 μg of salbutamol) and percentage of sputum eosinophils (p = 0.3645). Neither FeNO level (p = 0.281) nor blood eosinophil percentage/count (p = 0.6027; p = 0.1236; respectively) did not correlate with bronchodilator reversibility.

**Fig. 2:**
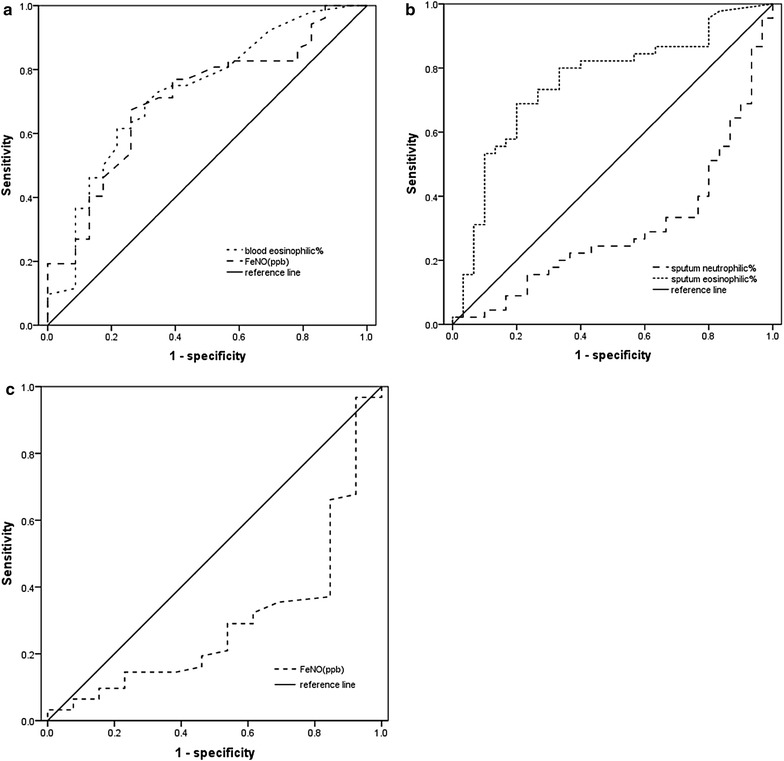
ROC curve for FeNO level and blood eosinophil percentage to predict sputum eosinophilia (≥ 2.5%) (**a**), ROC curve for sputum eosinophil/neutrophil percentage to predict FeNO level (≥ 32 ppb) (**b**), ROC curve for FeNO level to predict sputum neutrophilia (≥ 65%) (**c**)

The ROC curve analysis identified FeNO level as the best predictor for sputum eosinophilia with an area under the curve (AUC) of 0.707 (p = 0.004). The optimum cut-point for FeNO level was 35.5 ppb, and this yielded a sensitivity of 67.3%, a specificity of 73.9%. The percentage of blood eosinophils was also highly predictive with an area under the curve of 0.73 (p = 0.002) at a blood eosinophils cut-off of 1.5%, sensitivity and specificity were 61.5 and 78.3%, respectively (Fig. 2a). In addition, 4.36% was the best diagnostic cut-off value of percentage of sputum eosinophils for 32 ppb of FeNO level, with an ROC AUC of 0.755 (p < 0.001), sensitivity and specificity were 68.9 and 80%, respectively (Fig. 2b). Although the sputum neutrophil percentage was correlated with FeNO level, a ROC curve of these parameters did not show useful values (AUC = 0.297, p = 0.003, Fig. 2b; AUC = 0.295, p = 0.021, Fig. 2c). Percentage of blood eosinophils (p = 0.97) failed in the correlation of FeNO level.

There was no difference between blood eosinophil percentage (p = 0.089) and absolute blood eosinophil count (p = 0.14) in the prediction of sputum neutrophilia.

## Discussion

This study assessed the correlation of FeNO level, sputum eosinophils and blood eosinophils in initial diagnosis and uncontrolled asthma. All the people in the study were Chinese. FeNO level and percentage of blood eosinophils can accurately predict sputum eosinophilia. Percentage of sputum neutrophils was correlated with FeNO level and percentage of blood eosinophils, but not enough to be clinically useful. We also identified some additional findings, namely PD_20_/∆FEV_1_ association with FeNO level and percentage of sputum/blood eosinophils, which may reflect nothing.

There is a need to include simple and accessible biomarkers in the management of asthmatic airway inflammatory subtype, FeNO and blood eosinophils are markers of local and systemic eosinophilic inflammation, respectively. According to the literature, biomarkers of airway eosinophilic inflammation (FeNO, blood eosinophils and others, such as the sputum eosinophils) are well correlated with each other [14–16]. However, there is disagreement in the literature as to the value of FeNO level and sputum/blood eosinophils and there is no study following the diagnostic criteria of China [17, 18]. Few studies have used ROC to assess the ability of FeNO and peripheral blood eosinophils to detect airway eosinophilic phenotypes. Our results resolve these issues by showing that FeNO level and peripheral blood eosinophils were an excellent predictor of sputum eosinophilia in the clinically important subgroup of patients who received initial diagnosis and were uncontrolled asthma.

Following the 2016 recommendations of the Chinese National Guidelines on Diagnosis and Management of Cough, eosinophilic airway inflammation was defined as percentage of eosinophils ≥ 2.5% in induced sputum [11]. As shown in Table 4, FeNO level and peripheral blood eosinophils percentage/count were different. Although induced sputum has been considered the “gold standard” for airway inflammatory phenotypes, measurement of FeNO has achieved wide acceptance in routine clinical practice because it is easy to perform and readily available readout. The 2016 recommendation also suggested that a significant increase in sputum eosinophils ≥ 2.5% was considered if the FeNO level was ≥ 32 ppb [11]. As shown in Table 5, the percentage of sputum eosinophils was significantly different; however, the difference in peripheral blood eosinophil percentage and absolute count were not significant. According to the 2017 recommendation of GINA, which recently published an evidence-based clinical research guideline, eosinophil, such as in induced sputum or peripheral venous blood, can predict the risk of exacerbations [1]. We predicted that sputum eosinophilia was considered if the peripheral blood eosinophil percentage was ≥ 2%. As shown in Table 6, percentage of sputum eosinophils was different; however, the difference between FeNO in the two groups was not significant.

In clinical practice, there is a tendency to generalize the correlation between FeNO and percentage of sputum eosinophils, although the two methods are useful to assess the eosinophilic airway inflammation [19–21]. Our present optimal cutoff point (35.5 ppb) is similar to this optimal cut-off point (32 ppb) in the 2015 recommendation of China. These results showed that FeNO was to distinguish the patients with uncontrolled asthma with sputum eosinophilia from those without, thereby indicating its potential use as a diagnostic biomarker for eosinophilic asthma. However, there are confounding factors that may affect FeNO values in many cases. As a nitrate-rich diet or the contamination of nasal NO increase, and smoking or spirometry decrease FeNO, these factors should be avoided or taken into account when measuring FeNO [6]. The cutoff value has been a question in dispute because normal or low FeNO levels do not exclude the presence of disease. Optimal cutoff points by calculating sensitivity and specificity on an ROC curve to assess diagnostic biomarkers of eosinophilic airway inflammation may not be clinically applicable, given that their sensitivity and/or specificity is often suboptimal compared to that of reference standard tests [22]. Furthermore, FeNO has a limited value to assess the sputum neutrophilia, our study provided that sputum neutrophil percentage was correlated with FeNO level, a ROC curve of these parameters did not show useful values (AUC = 0.297, p = 0.003; AUC = 0.295, p = 0.021). Bronchial induced sputum cytology provides a more accurate approximation of airway inflammation phenotypes in asthma patients than FeNO.

The 2017 GINA recently published an evidence-based clinical research guideline that blood eosinophils may be a biomarker of exacerbation risk in patients with a history of exacerbation and can predict the effects of ICS on exacerbation prevention [1]. Because of their accuracy and convenience, blood eosinophils can be used in the clinic for detecting airway eosinophilia in uncontrolled asthma. One study in uncontrolled asthma has shown that peripheral blood eosinophil percentage (2.7%) and absolute count (0.26 × 10^9^/L) can serve as a diagnostic biomarker of sputum eosinophilia (≥ 3%) (AUC 0.907, sensitivity = 92.2%, specificity = 75.8%; AUC 0.898, sensitivity = 83.1%, specificity = 82.8%, respectively) [10]. These finding also indicates that percentage of blood eosinophils has a higher relevance than FeNO with eosinophil sputum in patients with asthma. These results suggest that blood eosinophils can be useful in assessing eosinophilic asthma and may have a role in selecting add-on therapy. However, there was no correlation between FeNO level and blood eosinophil percentage/count (p = 0.5801) in our study.

### Limitations and future research

One limitation of the study was that the p-value (p = 0.0477) changed slightly under 0.05, and the correlation coefficients (r = − 0.2294) were very low, as shown in Fig. 1d. The given number of data points may not be taken as strong evidence for such a relationship. It is possible that no relevant correlation between sputum neutrophil percentage and blood eosinophil percentage exists in the clinical practice.

The other was smoking cigarette factor. It is well known that smoking decreases FENO level [23]. Furthermore, some studies of the general population have reported that smoking increase blood eosinophils [24–26]. Other studies have reported blood eosinophils seems to be lower in asthmatic smokers than in asthmatic non-smokers [20, 27–29], and one study indicated that FeNO and blood eosinophils were significantly correlated in patients who have never smoked and former smokers but not in current smokers. There is disagreement in the literature as to the value of blood eosinophils [30]. Further investigation of smoking or no smoking factor in asthma would also benefit from bigger sample sizes.

## Conclusion

This study provides that inflammatory biomarkers, including sputum eosinophils, FeNO level and blood eosinophils, can accurately predict sputum eosinophilia in patients with uncontrolled asthma. It suggests that peripheral blood eosinophil is a useful tool better than FeNO level for monitoring sputum eosinophilia in uncontrolled asthma. FeNO level is poor surrogates for sputum neutrophils and blood eosinophils. These data may be useful for identifying patients with eosinophilic airway inflammation who will have a beneficial response to treatment with an ICS, and it is important to help guide treatment and management of asthmatic patients.

## Abbreviations

FeNO: fractional exhaled nitric oxide
BHR: bronchial hyper-responsiveness
GINA: the Global Initiative for Asthma
FVC: forced vital capacity
FEV1: forced expiratory volume in 1 s
PEF: peak expiratory flow
MMEF: maximum mid-expiratory flow
MEF: maximal expiratory flow
